# ‘Single-subject studies’-derived analyses unveil altered biomechanisms between very small cohorts: implications for rare diseases

**DOI:** 10.1093/bioinformatics/btab290

**Published:** 2021-07-12

**Authors:** Dillon Aberasturi, Nima Pouladi, Samir Rachid Zaim, Colleen Kenost, Joanne Berghout, Walter W Piegorsch, Yves A Lussier

**Affiliations:** Center for Biomedical Informatics and Biostatistics (CB2), University of Arizona Health Sciences, University of Arizona, Tucson, AZ, USA 85721; Department of Medicine, University of Arizona, Tucson, AZ, USA 85724-5035; Graduate Interdisciplinary Program in Statistics & Data Science, Graduate Interdisciplinary Program, University of Arizona, Tucson, AZ, USA 85721; Department of Medicine, University of Arizona, Tucson, AZ, USA 85724-5035; Department of Biomedical Informatics, University of Utah, UT, USA 84108; Center for Biomedical Informatics and Biostatistics (CB2), University of Arizona Health Sciences, University of Arizona, Tucson, AZ, USA 85721; Department of Medicine, University of Arizona, Tucson, AZ, USA 85724-5035; Graduate Interdisciplinary Program in Statistics & Data Science, Graduate Interdisciplinary Program, University of Arizona, Tucson, AZ, USA 85721; Center for Biomedical Informatics and Biostatistics (CB2), University of Arizona Health Sciences, University of Arizona, Tucson, AZ, USA 85721; Department of Medicine, University of Arizona, Tucson, AZ, USA 85724-5035; Graduate Interdisciplinary Program in Statistics & Data Science, Graduate Interdisciplinary Program, University of Arizona, Tucson, AZ, USA 85721; Department of Biomedical Informatics, University of Utah, UT, USA 84108; Center for Biomedical Informatics and Biostatistics (CB2), University of Arizona Health Sciences, University of Arizona, Tucson, AZ, USA 85721; Department of Medicine, University of Arizona, Tucson, AZ, USA 85724-5035; Ctr for Appl. Genetics and Genomic Medic, University of Arizona, Tucson, AZ, USA 85721; Center for Biomedical Informatics and Biostatistics (CB2), University of Arizona Health Sciences, University of Arizona, Tucson, AZ, USA 85721; Graduate Interdisciplinary Program in Statistics & Data Science, Graduate Interdisciplinary Program, University of Arizona, Tucson, AZ, USA 85721; Bio5 Institute, University of Arizona, Tucson, AZ, USA 85721; Center for Biomedical Informatics and Biostatistics (CB2), University of Arizona Health Sciences, University of Arizona, Tucson, AZ, USA 85721; Department of Medicine, University of Arizona, Tucson, AZ, USA 85724-5035; Graduate Interdisciplinary Program in Statistics & Data Science, Graduate Interdisciplinary Program, University of Arizona, Tucson, AZ, USA 85721; Department of Biomedical Informatics, University of Utah, UT, USA 84108; Ctr for Appl. Genetics and Genomic Medic, University of Arizona, Tucson, AZ, USA 85721; Bio5 Institute, University of Arizona, Tucson, AZ, USA 85721

## Abstract

**Motivation:**

Identifying altered transcripts between very small human cohorts is particularly challenging and is compounded by the low accrual rate of human subjects in rare diseases or sub-stratified common disorders. Yet, single-subject studies (S^3^) can compare paired transcriptome samples drawn from the same patient under two conditions (e.g. treated versus pre-treatment) and suggest patient-specific responsive biomechanisms based on the overrepresentation of functionally defined gene sets. These improve statistical power by: (i) reducing the total features tested and (ii) relaxing the requirement of within-cohort uniformity at the transcript level. We propose *Inter-N-of-1*, a novel method, to identify meaningful differences between very small cohorts by using the effect size of ‘single-subject-study’-derived responsive biological mechanisms.

**Results:**

In each subject, *Inter-N-of-1* requires applying previously published S^3^-type *N-of-1-pathways MixEnrich* to two paired samples (e.g. diseased versus unaffected tissues) for determining patient-specific enriched genes sets: Odds Ratios (S^3^-OR) and S^3^-variance using Gene Ontology Biological Processes. To evaluate small cohorts, we calculated the precision and recall of *Inter-N-of-1* and that of a control method (GLM+EGS) when comparing two cohorts of decreasing sizes (from 20 versus 20 to 2 versus 2) in a comprehensive six-parameter simulation and in a proof-of-concept clinical dataset. In simulations, the *Inter-N-of-1* median precision and recall are > 90% and >75% in cohorts of 3 versus 3 distinct subjects (regardless of the parameter values), whereas conventional methods outperform *Inter-N-of-1* at sample sizes 9 versus 9 and larger. Similar results were obtained in the clinical proof-of-concept dataset.

**Availability and implementation:**

R software is available at Lussierlab.net/BSSD.

## 1 Introduction

Empirical evidence unveils a methodological gap when comparing transcriptomic differences in biological mechanisms within very small human cohorts due to variations in heterogenicity, uncontrolled biology (age, gender, etc.) and diversity of environmental factors (nutrition, sleep, etc.) (Griggs *et al.*, 2009; Liu *et al.*, 2014; Schurch *et al.*, 2016; Soneson and Delorenzi, 2013). Even in isogenic conditions, studies recommend at least six biological replicates for applying GLMs (Liu *et al.*, 2014; Schurch *et al.*, 2016). These sample size requirements are unfeasible for clinical care of a patient. Paradoxically, rare diseases are common: 8% prevalence in the population (Elliott and Zurynski, 2015) and 26% of children who attend disability clinic (Guillem *et al.*, 2008). As timely and sizeable patient accrual of rare or micro-stratified diseases are prohibitive, there lies an opportunity for empowering clinical researchers with feasible statistical designs that enable smaller cohorts.

On the other hand, well-controlled isogenic studies (e.g. cellular models) can yield differentially expressed genes (DEGs) between two small samples. We and others have applied the power of the isogenic framework through the comparison of two sample transcriptomes from one subject in single-subject studies (S^3^). While *transcript-level* differences between two-sample remain inaccurate (Vitali *et al.*, 2019; Zaim *et al.*, 2019), *gene set-level (pathway/biosystem)* S^3^ have been shown to accurately discover altered biomechanisms from paired transcriptome samples drawn from the same patient under two conditions (e.g. tumor-normal, treated-untreated) (Ozturk *et al.*, 2018; Vitali *et al.*, 2019). The results of the S^3^  *gene set* analyses have been validated in various contexts such as cellular/tissular models (Balli *et al.*, 2019; Gardeux *et al.*, 2014, 2015), retrospectively in predicting cancer survival(Li *et al.*, 2017a,b; Schissler *et al.*, 2015; Schissler *et al.*, 2018) circulating tumor cells (Schissler *et al.*, 2016), biomarker discovery simulations (Zaim *et al.*, 2018) and therapeutic response (Li *et al.*, 2017a,b). Despite the success of these models to derive effect sizes and statistical significance in S^3^ of transcriptomes, these samples are isogenic or quasi-isogenic, and thus do not necessarily generalize to a group of subjects (*cohort-level signal)*. To address the latter, we reported that determining single cohort-level significance by combining gene set signal (e.g. pathways) from S^3^ analyses can be more accurate than conventional DEG analyses (e.g. GLMs) followed by gene set enrichment analysis (GSEA) (Subramanian *et al.*, 2005) in small cohort simulations (Zaim *et al.*, 2018) and in previously published datasets (Li *et al.*, 2017a,b). However, these methods could only summarize information within a single cohort and were not designed to compare two distinct cohorts to evaluate subgroup interactions.

To address the methodological gap, we therefore hypothesized that single-subject transcriptomic studies of gene sets increase the transcriptomic signal-to-noise ratio within subject and lead to an improved signal between small patient cohorts, as small as 3vs3 subjects per group. While technically different from the analysis of the standard two factor interactions in conventional cohort statistics, the proposed framework is conceptually related to a statistical interaction in that a within-single-subject analysis (subject-specific transcriptome dynamics) is followed by within-group agreement for characterizing Factor 1 (e.g. cancer versus paired normal tissue) and between group comparisons (Factor 2; e.g. responsive versus unresponsive to therapy). The strategy improves the statistical power by: (i) reducing the total features tested (gene set-level rather than transcript-level), (ii) relaxing the requirement of within-cohort uniformity at the transcript level as the coordination is conducted at the gene set-level and (iii) reducing confounding factors through the paired sample design of S^3^-analyses within subject. The novel bioinformatic method identifies meaningful biomechanism differences between very small cohorts by using single-subject-study-derived effect sizes for gene sets. Additionally, we show through both extensive simulations and a real data case example using TCGA human breast cancer cohort data that -within cohorts of varying sizes (3 to 7 subjects)- the *Inter-N-of-1* method outperforms traditional methods, which are based on generalized linear modeling (GLM) followed by common gene set enrichment or overlap analysis. We then apply this novel method to the effect sizes of two different single-subject analyses to illustrate the flexibility and utility of the proposed method for a variety of inputs.

## 2 Materials and methods

**Fig. 1.**
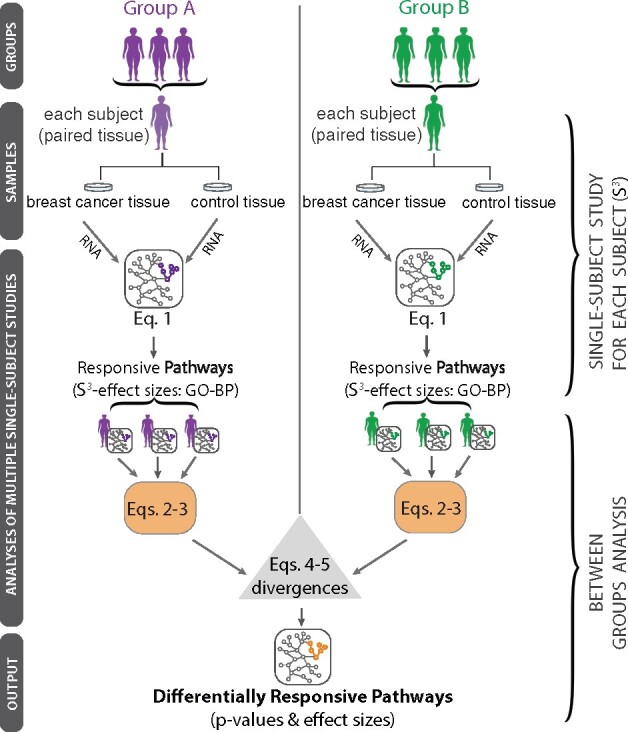
Overview of the gene set analyses (*Inter-N-of-1*) that leverage effect sizes and variances from S^3^ to conduct subsequent group comparisons. S^3^ details are provided in Figure 2

Figure 1provides an overview of the proposed new method (*Inter-N-of-1*). To motivate the development of transcriptome analytics between very small human samples, by nature heterogenicity, we first demonstrate the limitation of a Generalized Linear Model to DEGs between 23 TP53 and 19 PIK3CA breast cancer samples. Next, we describe two new methods *Inter-N-of-1* (MixEnrich) and *Inter-N-of-1* (NOISeq) that work by combining single subject study results using contingency tables to obtain cohort-level estimates for enrichment of GO terms which are then contrasted to discern differences in enrichment between the two cohorts. We then compare these two methods to a Generalized Linear Model (implemented in *LIMMA*) (i) in simulation studies with parameters estimated from empirical analyses of real datasets and (ii) in a proof-of-concept study of TCGA breast cancer cohorts. Also, the evaluation of the proposed new methods is conservative as it is conducted against a reference standard built with a distinct Generalized Linear Model *(edgeR*) using all samples.

### 2.1 Datasets

- We obtained 5179 g*ene sets* from Gene Ontology (Ashburner *et al.*, 2000) Biological Processes (GO-BP) (downloaded on 02/07/2019).
- The **Human breast cancer cohort** consists of RNA-seq expression profiles samples of 224 paired breast cancer (BC) tumor and unaffected breast tissue normal **(*Factor 1*)** from the same subjects (*n* = 112) from The Cancer Genome Atlas (TCGA) Breast Invasive Carcinoma data collection (Cancer Genome Atlas, 2012; Ciriello *et al.*, 2015) (Obtained 10/22/2015). The *proof-of-concept application* of the proposed methods (Figs 3 and 4) pertains to the subset of subjects with either *TP53* (n = 23) or *PIK3CA* (n = 19) mutations **(*Factor 2*)**, but not both. These BC oncogenes have been reported (i) in expression patterns (Cancer Genome Atlas, 2012), (ii) cancer subtypes (Van Keymeulen *et al.*, 2015), (iii) clinical outcomes (Kim *et al.*, 2017) and (iv) responsiveness to specific therapies (Andre *et al.*, 2019). These data were downloaded using the R package TCGA2STAT(n=42 cases; 84 files) (Wan *et al.*, 2016). **Data access and preparation:** (A) within each sample pair of a single-subject study, (i) we removed all transcripts with mean expression ≤5 counts, (ii) found the union of all genes remaining amongst all pairs, (iii) excluded all genes not included in the union of these two steps (17,923 genes remaining) and (iv) added ‘1’ to expression counts to eliminate ‘zeros’. (B) For GLM analyses, we eliminated all the transcripts with 0 counts for each subject and calculated each transcript’s coefficient of variation (CV). We retained the top 70% of transcripts ranked by CV (13,932 genes remaining).
- **Simulated datasets** production is described in Section 2.4.
- **MCF7 breast cancer cells,** for estimating simulation parameters, consist of 7 estrogen-stimulated and 7 unstimulated cells sample replicates (Edgar *et al.*, 2002; Liu *et al.*, 2014) (obtained on 10/14/2020; GEO GSE51403). 30M reads sequences of MCF7 cells were aligned using the hg19 (Dreszer *et al.*, 2012) reference genome and normalized RNA-seq counts into Fragments Per Kilobase of transcript per Million mapped reads.

### 2.2 Proposed S^3^-anchored responsive pathway *Inter-N-of-1* methods for comparing very small human cohorts

The following paragraphs will develop the methodology by which we conduct S^3^ prior to cross-cohort comparison to discover the effect size of responsive pathways in each subject and increase the features signal-to-noise ratio. Table 1 summarizes the variables.

**Table 1.** Variable definitions

Variable	Definition
$g_{gs,k_{j}}$	The number of DEGs within gene set gs for subject $k_{j}$ in cohort K
${g'}_{gs,k_{j}}$	The number of genes NOT differentially expressed in gene set gs for subject $k_{j}$ in cohort K
$h_{gs,k_{j}}$	The number of DEGs NOT in gene set gs for subject $k_{j}$ in cohort K
${h'}_{gs,k_{j}}$	Number of genes neither differentially expressed nor in gene set *gs* for subject $k_{j}$ in cohort K
N	Number of gene sets
$P\left(\cdot \right)$	Probability of Event (⋅) occurring
${\tilde{p}}_{gs,\Delta }$	*P*-value for gene set gs produced by the *Inter-N-of-1*
$Q_{gs,k_{j}}$	Continuity-corrected log S^3^-OR corresponding to gene set gs for subject $k_{j}\;$in cohort K
${\overset{-}{Q}}_{gs,K}$	The mean continuity-corrected log S^3^-OR in gene set gs in cohort K
$S_{K}$	The number of subjects in a cohort K (e.g. those with a PIK3CA or with TP53 somatic mutation)
$\theta_{K}$	Expected value of the continuity corrected log S^3^-OR for the molecular-defined cohort K
$\text{var}\left(Q_{gs,k_{j}}\right)$	Variance of continuity-corrected log S^3^-OR corresponding to gene set gs for subject $k_{j}$ in cohort K
$W_{gs,\Delta }$	The test statistic for the *Inter-N-of-1* for gene set gs
Z	A standard normal random variable

**Fig. 2.**
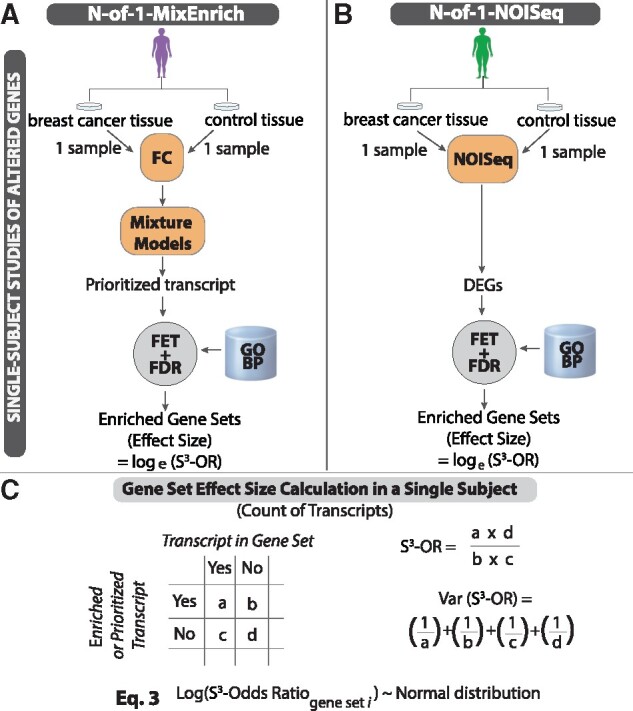
**Overview of two S^3^ methods conducted from one sample per condition without replicate generating effect sizes and variance for each gene set**. We apply S^3^ to each subject to identify either prioritized transcripts (**A**) or DEGs (**B**) between paired tumor-normal samples. We identify patient specific enriched gene sets and associated effect sizes in the form of natural log odds ratios through a FET (**C**). Each effect size is approximately normally distributed with known variance and mean, simplifying subsequent analyses between cohorts. The gene set-level variance enables the extraction of more information from each individual subject than typical variance estimators that work across subjects and thereby leads to increased statistical power. The N-of-1-MixEnrich method was previously described and validated (Berghout *et al.*, 2018; Li *et al.*, 2017a,b; Zaim *et al.*, 2018). NOISeq is also considered as an alternative meriting evaluation because of its performance in prior S^3^ evaluations (Zaim *et al.*, 2019)

**Identification of overrepresented gene sets for each subject:** As illustrated in Figure 2A, we applied to each of the tumor-normal pairs the N-of-1-*pathways* MixEnrich method that we had previously developed and validated (Li *et al.*, 2017a,b; Zaim *et al.*, 2018) and extended to account for direction of differential expression (Berghout *et al.*, 2018) and contrasted to other methods (Li *et al.*, 2019). Briefly, this method models the absolute value of the log_2_ transformed fold change (FC) for each gene across the two paired transcriptomes being studied and uses a probabilistic Gaussian mixture to assign a posterior probability that the gene is differentially expressed between tumor and normal conditions. Within the simulation, prioritized transcripts were defined as those with a posterior probability of being differentially expressed higher than 0.99. Within the TCGA breast cancer cohort, said definition included having both a posterior probability of being differentially expressed higher than 0.99 and an absolute-valued log_2_FC higher than log_2_(1.2), which was determined as optimal for downstream GO terms enrichment in this dataset. Genes were assigned to gene sets using the Gene Ontology (Ashburner *et al.*, 2000) Biological Process (GO-BP) hierarchy, filtered to those terms with gene set size between 15 and 500 genes, with subsumption to maximize interpretability. These DEGs were used to determine the overrepresented, or enriched, gene sets of interest using a two-sided Fisher’s Exact Test (FET) (Fisher, 1935) with a False Discovery Rate (FDR) of 5%. The output of this analysis generated lists of gene sets, with each list representing a single subject’s tumor-normal pair and comprising GO-BP terms accompanied by contingency table counts which were used to calculate an odds ratio (S^3^-OR) as the effect size.

We also applied NOISeq-sim to each of the tumor-normal pairs (Tarazona *et al.*, 2015) as shown in Figure 2B. NOISeq-sim simulates counts for each transcript under both conditions and then estimates the joint noise distribution for the |log_2_FC| and the | Δ(gene expression) | between conditions. The estimated noise distribution provides the probability of a DEG. For these applications of NOISeq with no replicates, the ‘*pnr*’ and ‘*v*’ parameters were set to 0.0002 and 0.00002 to prevent the method from producing any errors related to setting the size of the inherent multinomial distributions to an integer too large for R to handle. The criteria for identifying genes as differentially expressed for NOISeq were the same as those used for N-of-1-MixEnrich. As shown in Figure 2C, we subsequently used this information to construct contingency tables and calculate the natural log odds ratio for *Inter-N-of-1*. This process generated two different applications of *Inter-N-of-1*, N-of-1-MixEnrich and NOISeq, to conduct the single-subject analyses preceding the cohort comparison.

**Comparing enriched *Gene Sets* across distinct cohorts:** We first combined the data within two distinct cohorts into single statistics whose null reference distributions were at least approximately normal. These within-cohort statistics were contrasted via scaled subtraction in a manner reminiscent of the two-sample t-test to establish the difference in gene set enrichment between the two cohorts. Let gs∈{1,…,N} index the specific ***g****ene* ***s****et* being studied where *N* is the total number of gene sets, *k_j_* indexes a specific subject in cohort K composed of $S_{K}$ individuals with subjects numbered $j\in \{1,\ldots ,S_{K}\}$, and K∈{A,B} indexes a specific cohort. Let Δ signify quantities relating to the difference between the two cohorts.

The *Inter-N-of-1* analytics for combining information within a cohort considers the abstract contingency table shown as Table 2 where the cell counts are representative for the gene set indexed by *gs* and the subject indexed by $k_{j}$.

**Table 2.** Notation for 2 x 2 contingency table cross-classifying DEG status with gene set status

	DEG	Not DEG
Gene set *gs*	${\;g}_{gs,k_{j}}$	${g'}_{gs,k_{j}}$
Not Gene set *gs*	$h_{gs,k_{j}}$	${h'}_{gs,k_{j}}$

We obtain DEGs from the application of a chosen single-subject analysis method (either N-of-1-MixEnrich or N-of-1-NOISeq) for a specific gene set *gs* in individual *k_j_* of cohort K to fill out the contingency table with counts in the format shown in Table 2. We apply a continuity correction by adding 0.5 to each of the cells in the contingency table to provide a small-sample adjustment in the odds ratio (Agresti and Kateri, 2011). The natural log S^3^ OR, denoted as $Q_{gs,k_{j}}$, Equation 1, is approximately normally distributed with variance $\text{var}\left(Q_{gs,k_{j}}\right)$given in Equation 2 (Woolf, 1955).

(1) $$Q_{gs,k_{j}}=\ln\left(\frac{\left({\;g}_{gs,k_{j}}+\frac{1}{2}\right)\cdot \left({h'}_{gs,k_{j}}+\frac{1}{2}\right)}{\left(h_{gs,k_{j}}+\frac{1}{2}\right)\cdot \left({g'}_{gs,k_{j}}+\frac{1}{2}\right)}\right)\;$$

 

(2) $$\text{var}\left(Q_{gs,k_{j}}\right)=\frac{1}{\left({\;g}_{gs,k_{j}}+\frac{1}{2}\right)}+\frac{1}{\left({g'}_{gs,k_{j}}+\frac{1}{2}\right)}+\frac{1}{\left(h_{gs,k_{j}}+\frac{1}{2}\right)}+\frac{1}{\left({h'}_{gs,k_{j}}+\frac{1}{2}\right)}$$

We average the $Q_{gs,k_{j}}$ values within their respective cohorts to obtain the average ln ORs

(3) $${\overset{-}{Q}}_{gs,K}=\frac{1}{S_{K}}\sum_{j=1}^{S_{K}}Q_{gs,k_{j}\;}∼N\left(\theta_{K},\sum_{j=1}^{S_{K}}\frac{\text{var}\left(Q_{gs,k_{j}}\right)}{S_{K}^{2}}\right)$$

When the null hypothesis $H_{0}:\theta_{A}=E[\ln\left({\text{OR}}_{A}\right)]=E[\ln\left({\text{OR}}_{B}\right)]=\theta_{B}$ is true then

(4) $$W_{gs,\Delta }=\frac{{\overset{-}{Q}}_{gs,A}-{\overset{-}{Q}}_{gs,B}}{\sqrt{\text{var}\left({\overset{-}{Q}}_{gs,A}\right)+\mathrm{var}\left({\overset{-}{Q}}_{gs,B}\right)}}∼N\left(0,1\right)$$

at least approximately. The corresponding two-sided *P*-value for gene set *gs* is

(5) $${\tilde{p}}_{gs,\Delta }=2\cdot P\left(Z>\left|W_{gs,\Delta }\right|\right)$$

where Z represents a standard normal random variable. An FDR adjustment via the Benjamini–Hochberg method (Benjamini and Hochberg, 1995) is then applied to the ${\tilde{p}}_{gs,\Delta }$ across all the GO terms tested in the particular application. To ensure that the method positively identifies gene sets that are enriched in at least one of the cohorts, we set all FDR adjusted *P*-values to 1.0 if both cohort means of the log odds ratios are negative. This step ensures interpretable results since impoverished GO terms with significantly fewer-than-expected DEGs are not well understood from a biological context.

### 2.3 Description of the generalized linear models and application of *Inter-N-of-1* methods for small cohort comparison and their evaluation in the TCGA human breast cancer cohorts

**Generalized Linear Model (GLM) Designs*:*** For the cohort analyses, we applied a generalized linear model as implemented in *limma* (Smyth *et al.*, 2005). Preceding application of the GLM, we performed trimmed mean of M values (TMM) normalization (Robinson and Oshlack, 2010) on the data pre-processed for cohort analysis. We applied the voom normalization (Law *et al.*, 2014) via the *limma* function *voomwithQualityWeights* in R.

We used the three different designs described in Table 3 for these GLM-based analyses, which were called the simple design, the interaction design and GLM+EGS respectively. We blocked by subject for each of these GLM designs and all FDR adjustments of *P*-values were done using the Benjamini–Hochberg False Discovery Rate (FDR) method (Benjamini and Hochberg, 1995).

**Table 3.** Three experimental designs used for the generalized linear models

Name	Level	What is compared	Results
Simple	Transcript	TP53_Tumoral—PIK3CA_Tumoral	Figure 3A
Interaction	Transcript	(TP53_Tumoral—TP53_Normal) – (PIK3CA_Tumoral—PIK3CA_Normal)	Figure 3B
GLM+EGS	Gene set	Find DEGs using Interaction Contrast Enrichment via FET	Figures 4 and 5

*Note*: In the analysis of subsets of the TCGA human breast cancer cohorts, genes were declared differentially expressed if their abs(log_2_FC) > log_2_(1.2) and their FDR-adjusted *P*-value < 0.05. Within the simulation, genes were declared differentially expressed if their FDR-adjusted *P*-values < 0.05.

**Reference standard construction within TCGA human breast cancer cohort data using *edgeR* generalized linear model followed by gene set enrichment:** We chose to construct reference standards using all samples of the human breast cancer cohort to estimate accuracies of analyses of smaller sample size. After pre-processing for cohort analyses, we applied generalized linear models as implemented in the R software package *edgeR* (Robinson *et al.*, 2010) at FDR < 5% to the entire TCGA human breast cancer cohort data to construct three reference standards corresponding to the three designs discussed in Table 3. Each reference standard evaluated the analyses of the TCGA breast cancer cohorts (TP53 versus PIK3CA) and used the same filter thresholds for classifying transcripts as differentially expressed, which were designed to maximize the number of enriched GO terms in this dataset. In the GLM followed by enrichment of gene set (GLM+EGS), the prioritized interacting transcripts are followed by a FET at FDR < 5%.

**Subsampling of the TCGA Breast Cancer Cohort and application of GLM and *Inter-N-of-1* methods:** For each of the values$\;S_{A}=S_{B}=S\in \{2,3,4,5,\;7,\;8,9\}$ we ran 100 subsamples of the total cohorts where we randomly selected without replacement S subjects with *TP53* and S subjects with *PIK3CA*, without requiring non-redundancy of the random samplings. We applied the GLM+EGS method and the N-of-1-MixEnrich and NOISeq versions of the *Inter-N-of-1* method to each of the selected cohorts (TP53 versus PIK3CA). For each of the three methods, FDR < 5% adjustment of the *P*-values was done with respect to all 5179 GO terms tested.

**Fig. 3.**
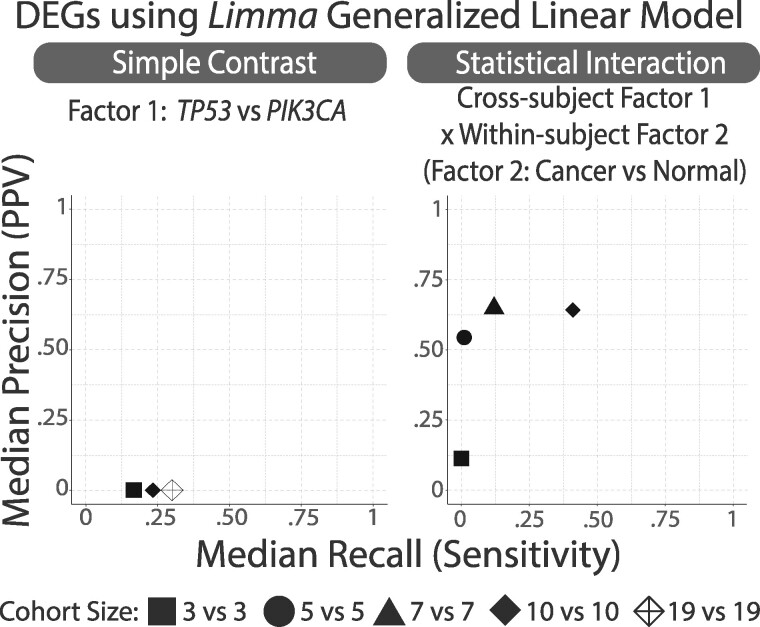
**At the transcript level, limited accuracies of Generalized Linear Models for calculating conventional simple contrast or interactions in small heterogenic breast cancer cohorts.** While GLMs can deliver DEGs in small cohorts for isogenic cellular and animal models, we recapitulate in the TCGA human breast cancer cohorts that small human cohorts are underpowered. We calculated the precision and recall scores associated with each of the 100 random sub-samplings of cohort sizes 2vs2, 3vs3, …, to 19vs19 for *TP53* versus *PIK3CA* and report median accuracie*s.* The left panel used a simple linear contrast of the tumor levels on the molecular subtypes. The right panel used a linear contrast corresponding to the interaction between the molecular subtypes (TP53 versus PIK3CA) and tumor status (Breast cancer versus normal breast). Discoveries were performed with *limma* while the reference standards were constructed with *edgeR*

For random subsamples of size $S_{A}=S_{B}=S\in \{2,3,4,\ldots 19$} of subjects, we applied the two transcript-level analyses using generalized linear models as implemented in *limma*. The performance of these transcript-level applications of *limma* was assessed and illustrated in Figure 3 to demonstrate the necessity and benefit of transforming from transcript-level to gene set-level analyses.

**Accuracy measures within TCGA human breast cancer cohorts:** For each method, we calculated the precision and recall using the following functions. When a method produced no positive predictions for the gene sets, we assigned values of zero to the precision and recall of the given method. Otherwise, we calculated the precision and recall using Powers' calculations with adjustments of adding 0.5 to numerators and 1.0 to denominators to avoid divisions by zero (Powers, 2011). In addition, we have previously published extensions to conventional accuracy scores that we termed ‘similarity Venn Diagrams’ and ‘Similarity Contingency Tables’ (Gardeux *et al.*, 2015). In these approaches true positive results between the prediction set and the reference standard include both identical GO-BP terms and those which are highly similar in terms of Information Theoretic Similarity (ITS) and therefore represent highly related biology (Tao *et al.*, 2007). We calculated the precision and recall of the gene set level analyses using this ITS approach because it unbiasedly compares predicted biological concepts against those of the reference standard. For precision, we included in the intersection those predicted GO-BP terms which had an ITS similarity of 0.70 or higher with any of the GO terms in the reference standards, while the denominator remained as all predicted GO-BP terms. Similarly, for recall, we included in the intersection the reference standard GO-BP terms which had an ITS similarity score of 0.70 or higher with any of the predicted GO terms, while the denominator remained as the total positive reference standard GO-BP terms. Of note, we previously reported that this ITS > 0.70 similarity criteria is highly conservative since ∼0.0056 pairs of GO-BP terms are similar at ITS > 0.7 (58,577 pairs among 10,458,756 non-identical combinations of GO-BP terms) (Gardeux *et al.*, 2015).

### 2.4 Simulation of small cohort comparisons to compare GLMs to *Inter-N-of-1* methods

**Data generation for simulation:** The overall scheme for the simulation began by constructing two cohorts of paired tumor-normal RNA-seq expression profiles. We calculated simulation parameters to most realistically create these expression values as described below (Table 4). To calculate statistical interactions between two factors, we had to design two cohorts of subjects and each subject with two sample conditions. We sought to recreate the TCGA breast cancer conditions with these parameters, using the observed median values in the TCGA dataset as the medians of the simulation parameters and varying the parameters around said medians. The TCGA dataset did not comprise repeated samples in the same condition, and thus we utilized the unstimulated MCF7 cell lines with seven replicates to estimate the variation expected between two paired normal tissues. In our previous pathway expression studies [(Yang *et al.*, 2012) and data not shown] where we compared two cohorts, about two-thirds of the observed responsive gene set patterns—as shown in Figure 2—consisted of a gene set responsive in one subject cohort and unresponsive in the other cohort.

**Table 4.** Simulation Parameter Values

Parameters	How Estimated	Values
Control Samples	Randomly sample without replacement from TCGA breast cancer normal samples	NA
log_2_FC distribution of non-differentially expressed genes	Calculate log_2_FCs of randomly paired MCF7 unstimulated breast cancer samples Split log_2_FCs into deciles by baseline expression All deciles containing 0 are combined into one category Sample with replacement from decile containing transcript name in first random pair	NA
Gamma parameters of log_2_FCs of DEGs	Run N-of-1-MixEnrich (Fig. 2) on within-subject tumor-normal pairs in TP53 and PIK3CA cohorts to identify DEGs MLEs for gamma parameters fit to absolute log_2_FCs of DEGs Used *egamma* function in *EnvStats R* package (Millard *et al.*, 2013)	Scale parameter = 6.06 Shape parameter = 0.55
Proportion of DEGs in enriched GO-BP terms	Split enriched GO terms from *edgeR* reference standard into deciles based on size Calculated DEGs median proportion for deciles containing GO-BP terms (size: 47, 200)	(GO size 200): 0.05, 0.10 (GO size 40): 0.10, 0.19
Proportion of Subjects with coordinated DEGs	Split log_2_FCs of DEGs within *edgeR* reference standard into categories >1.3, b) < -1.3 or c) neither Assign the max proportion of subjects per categories (a) or (b) for each transcript Find the median proportion of subjects across all transcripts	0.25, 0.48, 0.75
Balanced Cohort Size	NA	2, 3, 7, 10, 30
GO-BP terms	Enriched: GO:0002221 (200 genes) Enriched: GO:0000096 (47 genes) Control: GO:0006733 (196 genes) Control: GO:0090184 (41 genes)	NA

*Note*: Only cohort size and the proportion of subjects with coordinated DEGs were varied. All other parameters were held constant. 30 datasets were generated for each parameter configuration leading to a total of 450 datasets.

These paired tumor-normal samples represented within-subject samples were constructed to have a proportion of the transcripts with altered expression between the tumor and normal states. Through the use of randomly sampling without replacement, we generated the normal tissue samples for these pairs after filtering out all genes in the 112 TCGA breast cancer normal tissues, which were not present within the MCF7 breast cancer dataset (leaving 17,414 genes).

For each sampled normal breast tissue sample, we generated transcript expression for a paired breast cancer sample of that subject rather than sampling the corresponding breast cancer sample from the TCGA data. To produce a paired tumor expression value for a non-differentially expressed gene, we first followed the steps outlined in Table 4 to randomly generate empirical log_2_ Fold Changes (log_2_FC) and then we set the gene’s expression as the product of the gene’s paired normal expression and 2 raised to the exponent of the log_2_FC value. To generate the expression value for an altered transcript in a tumor sample, we randomly sampled a log_2_FC from a gamma distribution with parameters described in Table 4 and set said gene’s expression to the product of the gene’s normal expression and two raised to the exponent of the log_2_FC value. We generated only positive log_2_FCs for the DEGs to improve the *GLM's* ability to detect them as differentially expressed cross subjects. We specified a gamma distribution for these positive log_2_FCs since all the absolute-valued log_2_FC distributions we examined possessed significant right-skew.

We chose to evaluate the methods using the four GO terms described in Table 4. In simulation cohort A, two of these GO-BP terms would be seeded with altered transcripts, thus enriched, and two would serve as controls. In cohort B, none of the four GO terms were enriched, thereby setting up an interaction effect between the within-subject and between-subject factors. Within the two enriched GO terms in cohort A, we randomly selected the proportions of genes specified in Table 4 to have altered expression. We used Bernoulli random variables with probabilities of success outlined in Table 4 to designate subjects within cohort A, which would share all their randomly selected DEGs. The remaining subjects within cohort A had all their DEGs randomly vary across subjects. It was hypothesized that the percentage of subjects with shared altered transcripts would strongly influence the performance of the GLM+EGS method since *limma* assumes the presence of coordination of gene expression across subjects. Thus, we varied the expected proportion of subjects with shared DEGs within cohort A (0.25, 0.48, 0.75) using quartiles estimated from real datasets along with the sizes of the two cohorts (2, 3, 7, 10, 30) while holding all other parameters constant. These two varying parameters make up the core of the simulation since these determine how GLM+EGS performs relative to *Inter-N-of-1*, while all other parameters provide a background for operation. We generated 30 datasets for each parameter combination leading to a total of 450 datasets for our downstream simulations.

**Data preprocessing within simulation:** (A) For the GLM analyses, we preprocessed the simulated data by removing all genes with mean expression values less than 30 across all the simulated transcripts and subsequently added 1 to each of the expression counts. (B) For the single-subject analyses, we applied a three-stage pre-processing method in which we (i) removed all the transcripts with mean expression less than 30 within each sample-pair and (ii) found the union across all pairs of genes remaining and eliminated any genes not contained within. (iii) The remaining genes for the single-subject analyses then had 1 added to their expression counts to eliminate any remaining zeroes.

**Application of methods to simulated data:** The GLM+EGS and the two versions of the *Inter-N-of-1* method were applied to each of the generated datasets as described previously. The Benjamini–Hochberg False Discovery Rate (FDR) (Benjamini and Hochberg, 1995) adjustments of the *P*-values generated for each technique were performed with respect to only the 4 selected GO terms that were tested for each combination of dataset and method. GOBP terms were declared positive for a method if their associated FDR adjusted *P*-values for said method were below 0.05.

**Accuracy measures within the simulation:** To estimate the overall performance of each method within the simulation, we calculated the number of true positives, true negatives, false positives and false negatives occurring within the 2 enriched and 2 control GO terms across all 30 resampling of each combination of parameters. When any of the methods made no positive predictions for the gene sets, we artificially assigned values of 0 to the precision and recall of the given method. Otherwise, we calculated the precision and recall through the use of their traditional formulae (Powers, 2011). 30 accuracy scores are thus available for each combination of parameters for each GO term size (40 and 200).

## 3 Results

We showed that using a two-step process, where we first enrich the signal-to-noise ratio by applying S^3^-analyses to paired data in single-subjects before combining across subjects, can capture stable signal and yield results comparable to those in the reference standard, even as cohort size decreases. By contrast, traditional techniques for identification of gene set-level biological mechanisms that differentiate between two cohorts rapidly lose power and yield unreliable results as the sample size decreases below five subjects per cohort.

The transcriptomic analyses of TCGA human breast cancer cohorts in Figure 3 recapitulates that small human cohorts are particularly difficult to analyze using GLMs due to their heterogenic conditions and lack of controlled environment. Thus, small human cohorts present a stark contrast to isogenic controlled experiment cell lines or animal models where the high signal to noise ratio makes transcriptomic analyses possible for very small sample sizes. These unsurprising results provide the justification for the development of the proposed GLM+EGS and *Inter-N-of-1* methods conducted at the gene set level. They also attest to the intrinsic lack of signal within the TCGA human breast cancer cohorts for such transcriptomic analyses.

**Fig. 4.**
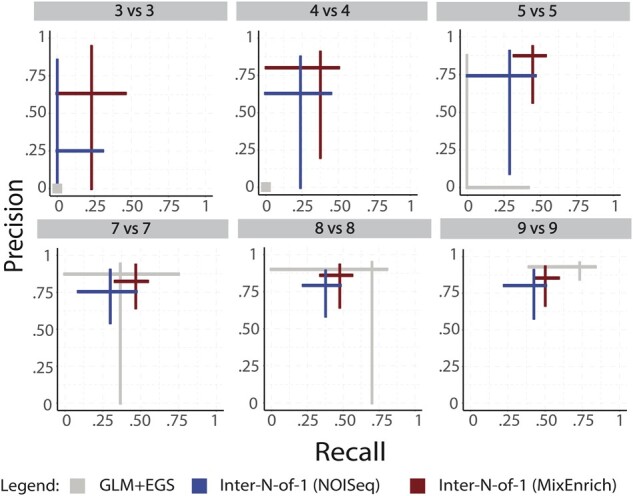
**At the gene set–level, two *Inter-N-of-1* methods outperform a GLM followed by enrichment in small heterogenic human cohorts.** While *Inter-N-of-1* (NOISeq) and *Inter-N-of-1* (MixEnrich) outperform the GLM followed by enrichment in gene sets for sample sizes ≤7vs7, the GLM+EGS shows better accuracy at sample sizes 9vs9 and above. Of note, GLM+EGS shows large variations in performance measures within the samples of size 8vs8 suggesting that despite its improved median accuracy it remains unreliable at that level. In all cases, the discovery of differentially responsive gene sets (*Inter-N-of-1* methods) or enriched gene sets (GLM+EGS) substantially outperform the accuracies of transcript-level analyses shown in Figure 3. While the *Inter-N-of-1* and GLM+EGS methods identify related signals, the reference standard designed by a distinct GLM+EGS approach favors the accuracies of the latter. In addition, *Inter-N-of-1* methods can assess the effect size of responsive gene sets in each subject, which can be illustrated as box plots of gene set response. In contrast. GLM+EGS methods are limited to a single description of over-representation calculated on interacting transcripts of the entire study. We calculated the precision and recall scores associated with each of the 100 random subsampling of cohort sizes 2vs2, 3vs3, 4vs4, 5vs5, 7vs7, 8vs8, 9vs9 for *TP53* and *PIK3CA* subjects with the GLM+EGS and *Inter-N-of-1* methods: (i) *Inter-N-of-1 (NOISeq)*, and (ii) *Inter-N-of-1 (MixEnrich)*. We also conducted a wide array of comparisons of unequal cohort sizes (not shown, e.g. 4vs8 mirrored results seen for 5vs5). The arms extend from the lower quartile to the upper quartile of the respective performance measure, and the two arms cross at the median for the precision and recall for that technique at the indicated cohort size

The performance results for subsets of the TCGA human breast cancer cohort data shown in Figure 4 establish that the two versions of the proposed *Inter-N-of-1* method degrade more gracefully in performance with decreasing cohort size than traditional GLM-based methods, thereby allowing them to outperform for smaller cohort sizes. Figure 4 shows that the niche where the *Inter-N-of-1* methods outperform in terms of median precision and recall extends to all cohort sizes below 7vs7, with the GLM+EGS method achieving higher median performance scores for 9vs9 and above. The sizes of the crosses suggest a further boon for the developed methods beyond this better ‘on average’ performance. The *Inter-N-of-1* methods tend to have very small tight crosses suggesting low variation in performance and greater consistency. The GLM+EGS method on the other hand possesses very large crosses until cohort size 9vs9, suggesting wild swings in performance across the different subsets evaluated. In addition, even the gene set-level GLM+EGS method outperforms transcript-level GLM analyses (Fig. 3 versus Fig. 4). Figure 4 also establishes that the N-of-1-MixEnrich version of the *Inter-N-of-1* method outperforms the NOISeq version in terms of consistency and median precision and recall. Although these differences remain small for larger cohort sizes of 7vs7 and above, they increase gradually with decreasing cohort sizes.

**Fig. 5.**
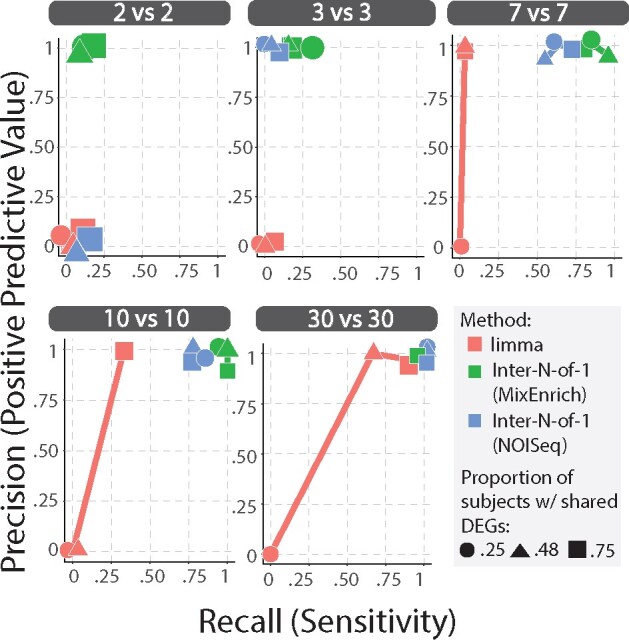
**Comparison of accuracy of GLM+EGS and *Inter-N-of-1* methods within the simulation.** We generated subject tumor-normal pairs for a variety of cohort sizes (2vs2, 3vs3, 7vs7, 10vs10, 30vs30) and expected proportion of subjects with shared DEGs in cohort A (0.25, 0.48, 0.75). We simulated 30 datasets for each parameter configuration and applied the proposed developed *Inter-N-of-1 methods* and GLM+EGS method to each. We calculated the total number of true positives, false positives, false negatives and true negatives across all iterations and used them to calculate the precision and recall for each combination of method, parameter configuration and GO term size. Separate graphs are made for each parameter configuration and plot the resulting precision and recall measures for each method for the gene sets of size 40 with Proportion of DEGs in enriched GO-BP terms = 0.10 (other proportions not shown). The results for gene sets of size 200 were very similar to the above results and so were excluded. The recall of the GLM+EGS rapidly declines for smaller cohort sizes, although increasing the expected proportion of subjects within cohorts with coordinated DEGs improves the recall of the method and decreases the minimum sample size needed for it to perform near perfectly

The simulations indicates that the proposed *Inter-N-of-1* methods outperform GLM+EGS for small sample sizes within parameters derived from cancer datasets and extended to investigate other conditions. Figure 5 shows that the two *Inter-N-of-1* methods are unaffected by changes in the expected proportion of subjects within cohorts with shared DEGs since their performance scores typically oscillate randomly around a fixed point given a fixed cohort size. These fixed points come closer to the perfect score of 1.0 precision and 1.0 recall with increasing cohort size, suggesting that mainly the cohort size affects the *Inter-N-of-1* method. The N-of-1-MixEnrich version of the *Inter-N-of-1* method generally performs the best out of all three methods, with its precision always staying 90% or higher and its recall typically reaching highest levels of any method. The NOISeq version of the *Inter-N-of-1* method suffers from a higher rate of false negatives for the two smallest tested cohort sizes of 2 and 3 and so displays significantly less recall than the N-of-1-MixEnrich version of the *Inter-N-of-1* method, although it does display similar levels of precision. Thus, this simulation also unveils the reason for which *Inter-N-of-1* (NOISeq) did not perform as well. Both cohort size and the expected proportion of subjects within groups with coordinated DEGs affect the performance of the GLM+EGS method. Increasing either of these parameters significantly improves the performance of the GLM+EGS method, with the single exception of the 2vs2 cohort size where GLM+EGS produces 0 precision and recall for all specifications of the proportion of subjects within group with coordinated DEGs. At the anti-conservative levels for these parameters, the GLM+EGS method matches the performance of the two versions of the *Inter-N-of-1* method. However, decreasing either parameter quickly leads the GLM+EGS method to underperform. For cohort sizes of 10vs10 and lower, the GLM+EGS method fails to match the performance of the two versions of the *Inter-N-of-1* method and so supports the superiority of *Inter-N-of-1* in such small sample sizes for breast cancer-like data.

## 4 Discussion

As stated in the introduction, empirical evidence suggests the existence of a methodological gap when comparing transcriptomic differences in biological mechanisms within very small human cohorts due to variations of heterogenicity, uncontrolled biology (age, gender, etc.) and diversity of environmental factors (nutrition, sleep, etc.). Yet, even in isogenic conditions, two studies have recommended at least six biological replicates for applying generalized linear models (Liu *et al.*, 2014; Schurch *et al.*, 2016; Soneson and Delorenzi, 2013). Examining two-factor interactions in transcriptomes (Cohorts × tumor status) further inflates the required sample size by a factor of 4 (Brookes *et al.*, 2004; Fleiss, 2004; Leon and Heo, 2009). Traditional cohort-based methods impose sample size requirements which simply cannot be met within the framework imposed by rare diseases, prompting the need to develop new methods. On the other hand, we and others have shown it is possible to obtain statistical significance of gene set-level effect size measures from single samples without replicates taken in two conditions, namely S^3^ (Li *et al.*, 2017a,b; Schissler *et al.*, 2015; Vitali *et al.*, 2019). We have shown evidence from studies of sampled human breast cancer cohorts and simulations that the S^3^-anchored *Inter-N-of-1* addresses this methodological gap. Their slow decay in performance when contrasted with the abrupt decay of GLM+EGS establishes the superiority of these methods for sample sizes of $S_{A}=S_{B}\in \{2,3,4,5,\;6\}\;$when applied to our TCGA human breast cancer cohort data. Comparison of the median precision and recall of the three considered techniques shows that on average our methods exhibit greater power and importantly less variable performance than GLM+EGS at these low cohort sizes. Our simulation study confirmed that both versions of the *Inter-N-of-1* provide substantially improved recall over the GLM+EGS method at small cohort sizes while still maintaining equivalent levels of precision. The simulation results also establish that the expected proportion of subjects with coordinated DEGs within cohorts plays a critical role in determining the range of cohort sizes in which the developed methods outperform traditional GLM-based techniques. In datasets where the proportion of subjects within cohorts sharing their DEGs is lower than 75%, the *Inter-N-of-1* methods continue to outperform the GLM+EGS method until cohort sizes >30. Furthermore, our applications of *Inter-N-of-1* to infrequent oncogenic mutations (TP53 versus PIK3CA) of human breast cancer cohorts (Cancer Genome Atlas, 2012) exemplifies how micro-stratified common disorders present a computational ad biological sampling problem related to those observed in infrequent diseases and a proxy for rare disorders.

Several limitations were observed. (i) This study focuses on parameters related to cancers, where substantial differences exist between paired normal and cancer tissues. While S^3^ have been shown to be effective in viral response (Gardeux *et al.*, 2015, 2017) or response to therapy (Li *et al.*, 2017a,b), it remains to be demonstrated that the downstream *Inter-N-of-1* methods can outperform transcript-level methods in those biological conditions. (ii) The simulation does present some inconsistencies with observations made within the TCGA human breast cancer subsets. Methods within the simulation attain performances which appear to be highly inflated compared to observations made within the small human cohorts. Part of this points to the need to alter simulation parameters to provide more realistic conditions. Part of these discrepancies can probably also be explained by the fact that the breast cancer analyses used a reference standard that favored GLM+EGS over *Inter-N-of-1* methods by design. (iii) We explored only one type of difference within gene set response between cohorts in the simulations: a cohort responsive versus unresponsive. We are thus undertaking the complementary analysis to compare the more general paradigm of gene sets more responsive in one cohort than in the other. (iv) Although the developed methods allow for a more accurate testing of interactions in datasets with small sample sizes, the importance of balancing confounders between the two cohorts cannot be overstated. The small samples used within these analyses prevent randomization from balancing key covariates and confounders between cohorts. Future studies could model unbalanced covariates through data or knowledge fusion with external datasets. (v) Transcript independence assumptions in the calculation of the single-subject odds ratio and its variance (*Inter-N-of-1* methods) may be transgressed. However, many such assumptions are routinely overlooked in related analyses, such as BH-FDR (Benjamini and Hochberg, 1995) with similar limitations later rectified as the BY-FDR (Benjamini and Yekutieli, 2001). Other methods for controlling FDR may offer increases in power, although these methods again may not properly control FDR under general dependence structures observed in gene expression data (Storey, 2002; Storey *et al.*, 2004). When viewed under that perspective, computational biology may progress by first proving new models and then addressing their biases in subsequent studies. (vi) Other unbiased approaches to generating gene sets could have been utilized (e.g. co-expression network from independent datasets, protein interaction networks, etc.). (vii) Of note, few datasets are available with two measures in different conditions per subject and more than one clinical cohort of subjects. Similar to physics where experimentalist and theory influence one another, our work presents improvements on solving an experimental design that is infrequently used and merits more consideration for increasing the signal-to-noise ratio in the study of rare and infrequent diseases. (viii) Prospective biologic validation of results is also required in future studies as we have done with S^3^ in the past (Gardeux *et al.*, 2014). (ix) The results presented for these methods are only for gene sets of size 15–500 and 40 and 200 (Simulation) and therefore validation of the performance of *Inter-N-of-1* in gene sets of larger size requires future work.

Another consideration concerns the fact that GLM+EGS and *Inter-N-of-1* evaluate different phenomena. The GLM+EGS method discovers GO terms enriched for transcripts and requires the coordination of signals at the transcript-level across subjects belonging to similar classes before the enrichment. The *Inter-N-of-1*, on the other hand, assesses whether the proportion of responsive transcripts within a given GO term measured in each subject significantly differs across cohorts. In other words, in the *Inter-N-of-1*, the transcripts contribution to the gene set signal may be different between subjects, while in the GLM+EGS methods a transcript-level coordination is required. The *Inter-N-of-1* favors clinical applications where gene set mechanisms are causal to the disease. Cancer is one such condition where numerous genetic and transcriptomic root causes may differ between subjects and yet converge to comparable cellular and clinical phenotypes.

In conclusion, the proposed S^3^-anchored *Inter-N-of-1*-methods demonstrate the utility of within-subject paired sample designs for better controlling the heterogenicity between subjects in a manner reminiscent of experimental isogenic models (e.g. cell lines or mice models). These results motivate further studies of new experimental designs, where paired within-subject samples allow analyses of datasets previously considered too small. The new design not only presents opportunities in terms of performance within small cohorts, but also in terms of utility. By examining the single-subject results one can directly see the degree of concordance and discordance amongst subjects and answer questions pertaining to whether specific subjects possess the overall observed signal. Thus, the *Inter-N-of-1* presented here represents not just a new method that performs better within small sample sizes, but also an example for how to simultaneously conduct analyses on patient variability within and across cohorts. In addition, precision therapies designed for increasingly sub-stratified common disorders can benefit from the proposed methods. The strategies and methods presented here open a new frontier that may greatly enrich our understanding of the genetic foundations of rare diseases.
